# K-Means Community Detection Algorithm Based on Density Peaks

**DOI:** 10.3390/e28020152

**Published:** 2026-01-29

**Authors:** Hongyan Gao, Jing Han, Yue Liu, Peng Zhang, Bo Yang, Yanqing Zu, Fei Liu, Yu Qian

**Affiliations:** 1 School of Physics and Opto-Electronic Technology, Baoji University of Arts and Sciences, Baoji 721016, China; bwlghy@163.com (H.G.); 17848579983@163.com (Y.L.); 18164294591@163.com (P.Z.); 17795463613@163.com (B.Y.); engreen74@163.com (Y.Z.); 2 Physics Teaching and Research Section, Fugu Middle School of Shaanxi Province, Yulin 719499, China; hhanj99@163.com

**Keywords:** complex network, community detection algorithm, D-means algorithm, density peak clustering

## Abstract

The identification of community structure is pivotal for understanding the functional characteristics of complex networks. To address the limitations of most existing community detection algorithms, which often require predefining the number of communities and lack robustness, this paper proposes a novel community detection algorithm named D-means (K-means community detection algorithm based on density peaks). This algorithm integrates the concept of density peak clustering with K-means spectral clustering, employing Chebyshev’s inequality to automatically determine the number of community centers, thereby enabling unsupervised identification of community quantities. By designing a multi-dimensional evaluation framework, the comparative experiments were conducted on LFR benchmark networks (Lancichinetti-Fortunato-Radicchi benchmark networks) and real-world social network datasets. The results demonstrate that the D-means algorithm outperforms traditional algorithms in terms of ACC (accuracy), ARI (adjusted rand index), and NMI (normalized mutual information) metrics, while also achieving improvements in runtime efficiency, showcasing strong robustness. Finally, the D-means algorithm was applied to the public transportation network of Urumqi. Empirical analysis identified 12 functionally significant transportation communities, providing theoretical support for urban rail transit optimization and commercial facility layout planning.

## 1. Introduction

Complex systems theory, at the forefront of systems science, profoundly reveals the universal patterns inherent in natural and social systems. It has become a focal point of interdisciplinary research across fields such as physics, mathematics, computer science, and transportation engineering [1,2,3]. As abstract models for describing complex systems, complex networks distill the commonalities of diverse systems into nodes and edges, thereby serving as powerful tools for characterizing and understanding real-world large-scale complex systems [4]. Within this framework, community structure—defined as subsets of nodes that are densely interconnected internally but sparsely connected externally—provides researchers with a mesoscale perspective situated between the macroscopic whole and microscopic individuals. This perspective is key to uncovering the functional modules and hierarchical organization inherent in complex networks [5]. For instance, analyzing the community structure of urban transportation networks can identify highly synergistic traffic subzones, offering crucial theoretical insights for optimizing traffic flow, planning infrastructure, and deploying commercial services [6].

In response to the highly heterogeneous structural characteristics of real-world networks, community detection methods have evolved into two main paradigms: global and local approaches. Global methods (e.g., the Louvain algorithm, spectral clustering) require complete network topological information and achieve network partitioning by optimizing global objective functions such as modularity [7]. These methods can generate globally consistent structural partitions but suffer from resolution limits and high computational complexity, making them difficult to apply directly to ultra-large-scale networks or dynamically evolving scenarios. The traditional K-means clustering algorithm, which optimizes by minimizing the sum of squared distances from nodes to cluster centers, is also widely used in community detection [8]. To enhance its adaptability to network data, researchers have proposed various improvements: for instance, Hajij et al. identified initial cluster centers using local PageRank values to improve stability [9]; Sommer et al. developed an autonomous kernel K-means framework that deeply integrates kernel techniques with modularity criteria [10]; Cai et al. introduced a Density-Degree (DD) index that combines node density and degree centrality to construct an improved K-means community detection framework [11]. However, most of these improved methods still rely on multiple iterative evaluations to determine the optimal cluster centers, a process that incurs high computational costs and significantly limits efficiency in large-scale network scenarios.

Concurrently, significant progress has been made in deep learning-based community detection methods, with graph neural network technology enabling effective joint modeling of network topology and node attributes. For instance, Kojaku et al. proposed a community detection method based on neural network embedding [12]; He et al. designed a semi-supervised overlapping community detection model named SSGCAE using graph neural networks [13]; and Guo et al. introduced a community detection algorithm based on adaptive graph contrastive learning [14]. However, such methods typically rely on large amounts of annotated data for training and suffer from a lack of interpretability in their decision-making processes, which to some extent restricts their broader application in the analysis of practical complex systems.

Local community detection methods, such as the LFM algorithm(local fitness maximization algorithm) and seed expansion algorithms, rely solely on local network information. They start from seed nodes and gradually expand to form communities. These methods offer advantages in computational efficiency and scalability, particularly excelling at identifying small-scale community structures. In recent years, local methods have continued to evolve. For example, Baltsou proposed a Hint Enhancement Framework that strengthens the importance of specific seed nodes through edge re-weighting or network rewiring strategies, followed by performing local community detection on the optimized network [15]. Meanwhile, Ni et al. developed the SLSS algorithm, a semi-supervised approach based on structural similarity measures, which requires only partial known community information to complete the detection task [16].

However, local methods still face significant limitations: their results are highly sensitive to the selection of seed nodes and often lack structural consistency from a global perspective. A systematic comparison reveals fundamental differences between global and local methods in terms of detection mechanisms and application scenarios—global methods tend to produce partitions with better structural consistency, while local methods hold a clear advantage in efficiency for large-scale network analysis.

An important trend in current research lies in integrating the strengths of both approaches. By combining global structural information with local computational efficiency, this fusion paradigm aims to enhance the quality of community partitions while maintaining scalability. Such integrated approaches are becoming crucial for addressing the challenges of complex network analysis.

Against this background, the Density Peaks Clustering (DPC) algorithm has garnered widespread attention due to its unique ability to automatically identify cluster centers [17]. By calculating the local density of data points and their distance to points of higher density, the algorithm enables intuitive selection of cluster centers through a two-dimensional decision graph. Since its introduction in 2014 [18], researchers have proposed various improvements to address its limitations in handling high-dimensional data, parameter sensitivity, and density heterogeneity [19,20,21].

Although the algorithm has achieved significant success in traditional data clustering, its application in complex network community detection remains exploratory. It faces two core challenges: first, how to directly process graph-structured data; and second, how to appropriately define “density” and “distance” metrics within a network environment. Currently, only a few studies have attempted to adapt this algorithm for network analysis, such as graph-based label propagation methods, which assign labels to remaining nodes to form final clusters [22].

In summary, two notable issues emerge from current research: On the one hand, there is an urgent need in the field of community detection for novel methods that can overcome the sensitivity of traditional K-means algorithms to initial centers and their reliance on multiple evaluations. On the other hand, although the DPC algorithm demonstrates significant advantages in automatically identifying centers, its application in community partitioning for complex networks remains very limited, particularly lacking in-depth analysis of multimodal transportation networks from the mesoscale perspective of community structure. It is worth noting that existing research still requires further exploration in the following areas: (1) how to effectively integrate the stability of global methods with the efficiency of local methods; (2) how to adapt advanced algorithms from the data clustering domain to network analysis scenarios; and (3) how to apply community detection methods to practical complex systems such as multimodal transportation networks.

Based on this, this study proposes a novel community detection algorithm that integrates Density Peaks Clustering with K-means. The core contributions of this research are: (1) leveraging the automatic center identification mechanism of the DPC algorithm to fundamentally address the initial center selection problem in K-means; (2) designing density and distance metrics suitable for complex network graph data, establishing an effective pathway for migrating data clustering algorithms to the network analysis domain; (3) applying the proposed algorithm to multimodal transportation networks to reveal their organizational patterns and operational mechanisms from the novel mesoscale perspective of community structure, providing scientific decision support for transportation system planning and management; and (4) conducting systematic experimental validation across multiple scenarios—including synthetic networks, social networks, and transportation networks—comprehensively comparing the proposed algorithm with traditional methods, local methods, and the latest hybrid methods to fully demonstrate its effectiveness and superiority.

## 2. Density Peak Clustering Algorithm

The DPC algorithm is a classical density-based clustering approach that detects density peak points as cluster centers by computing the local density and relative distance of each data point, then assigns the remaining points to their corresponding clusters. As an innovative clustering algorithm, DPC is capable of identifying non-convex clusters and relies on two crucial parameters: the distance parameter and the density threshold. The core idea of this algorithm is based on the following two fundamental assumptions:

**Assumption** **1.** *Cluster centers are characterized by higher local density compared to surrounding points.*

**Assumption** **2.** *The distance between different cluster centers in all clusters is relatively large.*

To mathematically formalize these two assumptions, consider a clustering dataset $X=\{x_{1},x_{2},\ldots ,x_{n}\}$. For each data point $x_{i}$, two key metrics are introduced, i.e., the local density $\rho_{i}$ and relative distance $\delta_{i}$, as described by the following definitions (Definitions 1 to 5).

**Definition** **1.** *The Euclidean distance is employed to measure the distance between any two data points in the dataset, calculated as follows:*

(1) $$dist(x_{i},x_{j})=\sqrt{\sum_{l=1}^{m}{\left(x_{il}-x_{jl}\right)}^{2}},$$

*where* dist(⋅,⋅) *denotes the Euclidean distance,* m *is the dimension of the data sample, and* $x_{il}$ *is the value of* $x_{i}$ *in the* l *th dimension.*

**Definition** **2.** *When applying the truncated density estimation method, the local density of a point i is determined by counting the number of neighboring samples that fall within its cutoff distance (also called the truncation radius). The mathematical expression is as follows:*

(2) $$\rho_{i}=\sum_{x_{j}\in X}\chi (dist(x_{i},\;x_{j})-d_{c}),$$

(3) $$\chi (x)={\{}_{0,\;\;x\geq 0,}^{1,\;\;x<0,}$$

*where* $\rho_{i}$ *is the local density,* $dist(x_{i},x_{j})$ *is the Euclidean distance between the sample* $x_{i}$ *and* $x_{j}$ *, and* $d_{c}$ *is the truncation distance of the data points.*

**Definition** **3.** *Using Gaussian density estimation, a point’s local density is calculated by summing the Gaussian-weighted distances from all other points, formulated as:*

(4) $$\rho_{i}={\sum_{x_{j}\in X}e^{-\left[\frac{dist(x_{i},x_{j})}{d_{c}}\right]}}^{2},$$

* where* $d_{c}$ *and* dist(⋅,⋅) *is consistent with the definition using truncated density estimation method.*

**Definition** **4.** *Relative distance* $\delta_{i}$ *refers to the minimum distance between sample* i *and other points with higher density values, written as:*

(5) $$\delta_{i}={\{}_{\underset{j}{\max}(dist(x_{i},\;x_{j})),\;otherwise,}^{\underset{j:\rho_{i}<\rho_{j}}{\min}(dist(x_{i},\;x_{j})),\;if\exists j\;s.t.\;\rho_{i}<\rho_{j},}$$

*The clustering performance varies with dataset size: the truncated kernel method demonstrates superior efficiency for large-scale datasets, while the Gaussian kernel approach yields more precise clustering results for smaller datasets.*

**Figure 1:**
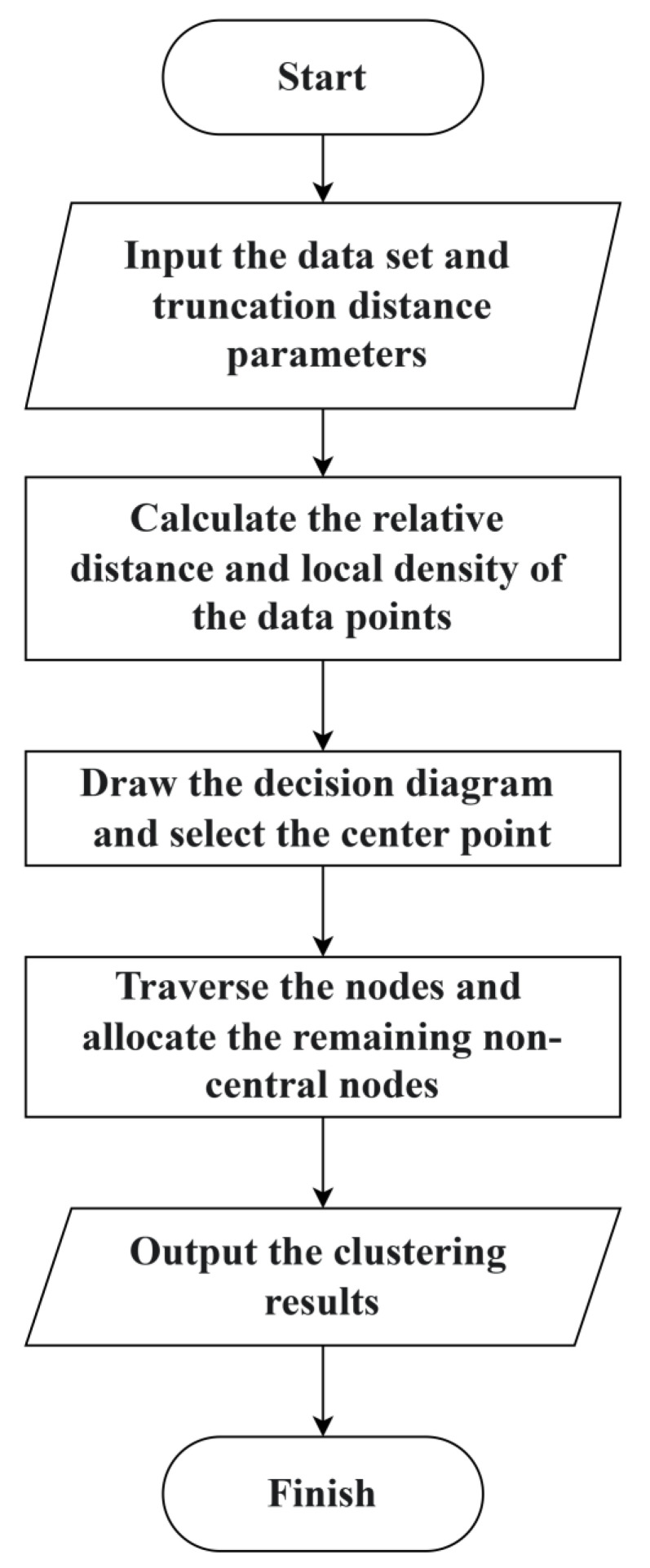
Flow Chart of Density Peak Clustering Algorithm.

The DPC algorithm operates without requiring pre-defined cluster centers. Instead, it automatically identifies optimal cluster centers through two key metrics: local density (ρ) and relative distance (δ). The fundamental principle involves detecting density peaks—data points that simultaneously exhibit both high local density and significant separation distance from any points of greater density. The implementation process involves: (1) computing ρ and δ values for all data points, (2) generating a decision graph with ρ on the horizontal axis and δ on the vertical axis, and (3) visually selecting cluster centers as distinct points occupying the upper-right region of the graph where both ρ and δ values are maximized. The complete algorithmic workflow is illustrated in Figure 1, with detailed execution steps outlined below:

Input: Dataset $X=\{x_{1},x_{2},\ldots ,x_{n}\}$, truncation distance parameter $d_{c}$.

Output: Clustering results $C=\{c_{1},c_{2},\ldots ,c_{m}\}$.

Step 1: Calculate the distance between each pair of data points based on the sample dataset X to obtain the distance matrix $D^{n\times n}$;

Step 2: Use the definition to calculate the local density $\rho_{i}$ and relative distance $\delta_{i}$ of each point $x_{i}$;

Step 3: Draw a decision diagram of $\left(\rho_{i},\delta_{i}\right)$ and visually select points with high local density and large relative distance values as the cluster center points;

Step 4: Traverse the nodes and assign each non clustered center data point to a cluster with a higher density and the closest distance to it, and finally output the clustering result.

**Figure 2:**
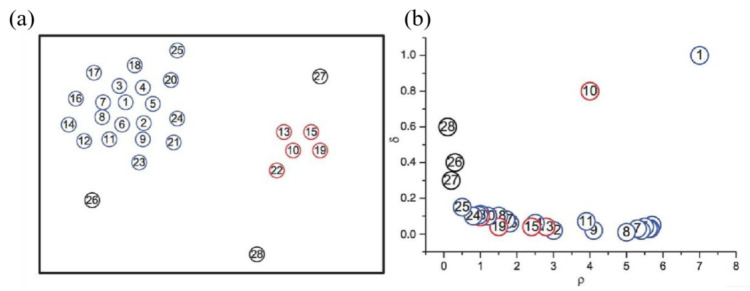
Dataset distribution and decision diagram: (**a**) Data distribution; (**b**) Decision diagram (Serial numbers represent node identifiers).

To illustrate the cluster center selection process using a decision graph, we present a demonstrative example in Figure 2, in which red and blue dots represent two different clusters, and black dots represent noise points. Figure 2a shows the distribution of 28 data points, and Figure 2b displays the corresponding decision graph (local density on the horizontal axis and relative distance on the vertical axis).

Cluster centers are selected from the decision graph by identifying points that simultaneously exhibit both high local density (ρ) and large relative distance (δ), as exemplified by points 1 and 10 in Figure 2b. These points appear in the upper-right region of the decision graph, representing optimal cluster center candidates. Conversely, points 26–28 demonstrate an inverse pattern with relatively large δ values but negligible ρ values, which characterizes them as noise points rather than valid cluster centers.

While the decision graph provides an intuitive approach for cluster center selection, this manual method suffers from subjectivity and inefficiency, particularly when processing complex datasets where visual identification becomes both time-consuming and error-prone. To address these limitations, we propose an automated selection criterion based on the product of local density and relative distance ($\gamma_{i}=\rho_{i}*\delta_{i}$). By sorting all data points in descending order of their γ values, the algorithm can automatically identify potential cluster centers as points with the highest γ scores. Following center selection, remaining points are efficiently assigned to their nearest higher-density neighbor in a single pass. This optimized version of the DPC algorithm achieves clustering in $O(n^{2})$ time complexity (where n represents the total data points) without requiring iterative computations, offering significant advantages in both automation and computational efficiency.

The DPC algorithm overcomes traditional clustering limitations by leveraging local density and relative distance metrics, yet it faces several critical challenges: its performance is highly sensitive to the truncation distance parameter ($d_{c}$) whose selection currently relies on empirical estimation rather than automated methods, introducing operational complexity; its robustness is compromised by noise and outliers due to potential similarities in density-distance characteristics between noise points and genuine cluster centers; and it suffers from the curse of dimensionality in high-dimensional spaces where conventional distance metrics become ineffective. To enhance the algorithm’s reliability and applicability, researchers have proposed various improvements including automated parameter selection techniques, advanced density measurement methods, and optimized allocation strategies to address these inherent limitations.

## 3. K-Means Community Detection Algorithm Based on Density Peak

Current research on similarity-based merging algorithms faces two primary challenges: determining the initial number of communities and optimizing the search strategy. The K-means algorithm, for instance, is highly sensitive to this initial configuration, which is often difficult to specify for real-world networks and significantly impacts the final clustering quality. Therefore, accurately and efficiently estimating the number of communities is crucial for performance.

The Density Peak Clustering algorithm addresses this by automatically determining the number of clusters based on data distribution, without requiring pre-specification. Inspired by this advantage, we propose a community detection algorithm that utilizes center and neighbor node information, adopting the core idea of automatically identifying community centers from density peaks.

However, the traditional density peak algorithm relies on coordinate information to compute local density and relative distance, making it unsuitable for network data. Thus, adaptations are necessary to better suit community detection in complex networks.

### 3.1. Density and Distance Attributes of Nodes

In complex network analysis, node density is a comprehensive metric. Its definition incorporates both the number of directly connected neighbors and the tightness of connections among those neighbors. This tightness is quantified by the number of edges between neighbors, referred to as the clustering coefficient. A higher node degree generally implies greater influence in the network, while a higher clustering coefficient reflects a stronger ability to aggregate surrounding nodes. Therefore, by integrating the leadership and cohesion of nodes, this paper proposes a local density index. A higher node density value indicates that the node is not only a key member within its community but is also surrounded by other significant nodes.

**Definition** **5.** *The density attribute of a node is defined as the sum of its degree and its clustering coefficient. For a node* i *, it is calculated as:*

(6) $$\rho_{i}=K_{i}^{\prime }+C_{i}^{\prime },$$

*where* ${K_{i}}^{\prime }$ *denotes the normalized degree of node* i *, and* ${C_{i}}^{\prime }$ *represents its normalized clustering coefficient. Due to the disparate scales where node degree can be much larger than 1 while the clustering coefficient is confined to [0, 1], we employed min-max normalization to scale both the node degree and the clustering coefficient into the [0, 1] interval. This metric relies solely on the network’s topological structure, thus effectively circumventing the high-dimensional challenges often associated with traditional density peak clustering algorithms.*

The distance attribute of a node is another crucial metric for identifying central nodes, in addition to its density attribute. In the proposed community detection algorithm, this attribute is derived by comparing density attributes between node pairs. For any two nodes i and j satisfying the following condition:

(7) $$\rho_{j}>\rho_{i}$$

(8) $$\rho_{j}=\rho_{i},\;C_{j}>C_{i},$$

**Definition** **6.** *The distance attribute of a node* i *is defined as the minimum value among the shortest path distances from this node to all others that possess a higher density attribute. This is formally expressed as:*

(9) $$\delta_{i}=\min(d_{ij}^{\prime }),$$

*where* $\delta_{i}$ *represents the distance attribute of node* i *, and* $d_{ij}^{\prime }$ *denotes the shortest path distance between nodes* i *and* j *, respectively.*

The calculation of a node’s distance attribute follows a hierarchical proximity-based search:

1. If any direct neighbor of node i possesses a higher density attribute, the distance attribute $\delta_{i}$ is assigned a value of 1.
2. If no higher-density node exists among direct neighbors, the search extends to second-degree neighbors (neighbors of neighbors). Should any second-degree neighbor have a higher density attribute, $\delta_{i}$ is set to 2.
3. If no node with higher density is found among either direct or second-degree neighbors, $\delta_{i}$ is set to 3.

Real-world networks typically exhibit small-world characteristics, characterized by short average path lengths and high clustering coefficients. As a result, distance attribute values $\delta_{i}$ in such networks are generally much smaller than density attribute values $\rho_{i}$. To ensure that both attributes contribute equally in the selection of community centers, Min-Max normalization is applied to scale each attribute to the interval [0, 1]. The normalization formula is as follows:

(10) $${\rho_{i}}^{nor}=\frac{\rho_{i}-\min(\rho )}{\max(\rho )-\min(\rho )},$$

(11) $${\delta_{i}}^{nor}=\frac{\delta_{i}-\min(\delta )}{\max(\delta )-\min(\delta )},$$

where ${\rho_{i}}^{nor}$ and ${\delta_{i}}^{nor}$ denote the normalized density attribute and normalized distance attribute of node i, respectively.

### 3.2. Selection of Community Centers

Nodes with higher centrality values are more likely to be community centers. To systematically select these central nodes, this paper employs the product index γ from the density peak clustering algorithm, which is computed as:

(12) $$\gamma_{i}=\rho_{i}^{nor}\times \delta_{i}^{nor},$$

Following the computation of the product index γ for all nodes, the values are sorted in descending order, and an upper limit is established using Chebyshev’s inequality. As a statistical theorem that does not assume any specific probability distribution, Chebyshev’s inequality provides a conservative upper bound for the probability that a random variable deviates from its expected value. The inequality is premised on the following condition:

**Theorem** **1.** *Let the random variable X have a mathematical expectation* μ*, and a variance *$\sigma^{2}$*. For any *ε>0*, satisfy the following conditions:*

(13) $$P\left\{\left|X-\mu \right|\geq \varepsilon \right\}\leq \frac{\sigma^{2}}{\varepsilon^{2}},$$

*Therefore, given a known mean *μ* and variance *$\sigma^{2}$*, a conservative upper bound for the probability of the random variable X can be derived. This upper bound does not depend on any specific probability distribution and is solely related to a single parameter *ε*. The process of selecting cluster centers from the decision graph reveals that the local density and relative distance of these centers exhibit a significant jump in the graph, whereas ordinary data points display low density and a clustered distribution. This observed pattern aligns with the principles of Chebyshev’s inequality. Since the probability distribution of the product index *γ* is determined by the value of parameter *ε*, an effective upper bound can be established for *$\gamma_{i}$* for the majority of data points.*

(14) $$|\gamma_{i}-\mu |\leq \varepsilon \sqrt{\sigma^{2}},$$

From the above equation, it can be inferred that if the distance from the $\gamma_{i}$ of a node i to the mean is greater than this value, the node is identified as a low probability data point, which is the central node, satisfying the following formula:

Based on the equation above, it can be inferred that if the deviation of a node i’s product index $\gamma_{i}$ from the mean exceeds this threshold value, the node is classified as a low-probability data point and identified as a central node. This condition satisfies the following expression:

(15) $$\gamma_{i}>(\mu +\varepsilon \times \sigma ),$$

where μ and σ represent the mean and standard deviation of the product index across all nodes, respectively.

As mentioned earlier, a major challenge of the traditional K-means algorithm in the process of community detection is the inability to automatically determine the number of communities, and the choice of community quantity directly affects the final quality of segmentation. This article uses density peak clustering algorithm to select an appropriate number of density peak points as community centers, where the ε need to be determined. The community centers are determined by ε, and the specific number of communities is also determined.

The D-means algorithm excels in achieving a balance between parametric simplicity and robustness of results. Unlike the K-means algorithm, which requires a predefined number of communities, or the Label Propagation Algorithm (LPA), which is sensitive to random initialization, the core hyperparameter of D-means is typically the neighborhood radius ε. This parameter intuitively defines the physical scale of node neighborhoods in the feature space.

Through systematic parameter sweep experiments, we observe that the performance of the D-means algorithm exhibits a typical three-phase pattern as ε varies:

Under-partitioning region (ε > ε_high): When ε is excessively large, the algorithm tends to merge multiple natural communities, resulting in a significantly lower number of communities than the ground truth. Metrics such as modularity (Q) and normalized mutual information (NMI) decline sharply.

Stable plateau region (ε_low ≤ ε ≤ ε_high): A broad interval exists for ε values. Within this range, the core community structure identified by the algorithm remains stable, with key performance indicators (e.g., ACC, NMI) fluctuating by less than 5%. This interval can typically be estimated efficiently using statistical measures of pairwise node distances, such as the 15th to 60th percentiles of the distance distribution, significantly reducing the difficulty of parameter tuning.

Over-partitioning region (ε < ε_low): When ε is too small, node neighborhoods become overly constrained, leading the algorithm to identify each node or its small clusters as independent communities. This results in a large number of trivial communities, rendering the partitioning results meaningless at a macroscopic level, while modularity also performs poorly. We set ε within the range of the 15th to 60th percentiles of the distance distribution.

### 3.3. Specific Execution Steps of D-Means Algorithm

This paper proposes a novel algorithm named the D-means algorithm. Based on the topological connections within the network structure, the algorithm assigns density and distance attributes to each node. Candidate central nodes are identified as those exhibiting higher density and greater distance values. The Chebyshev inequality is then employed to automatically select potential central nodes from the candidate set.

**Figure 3:**
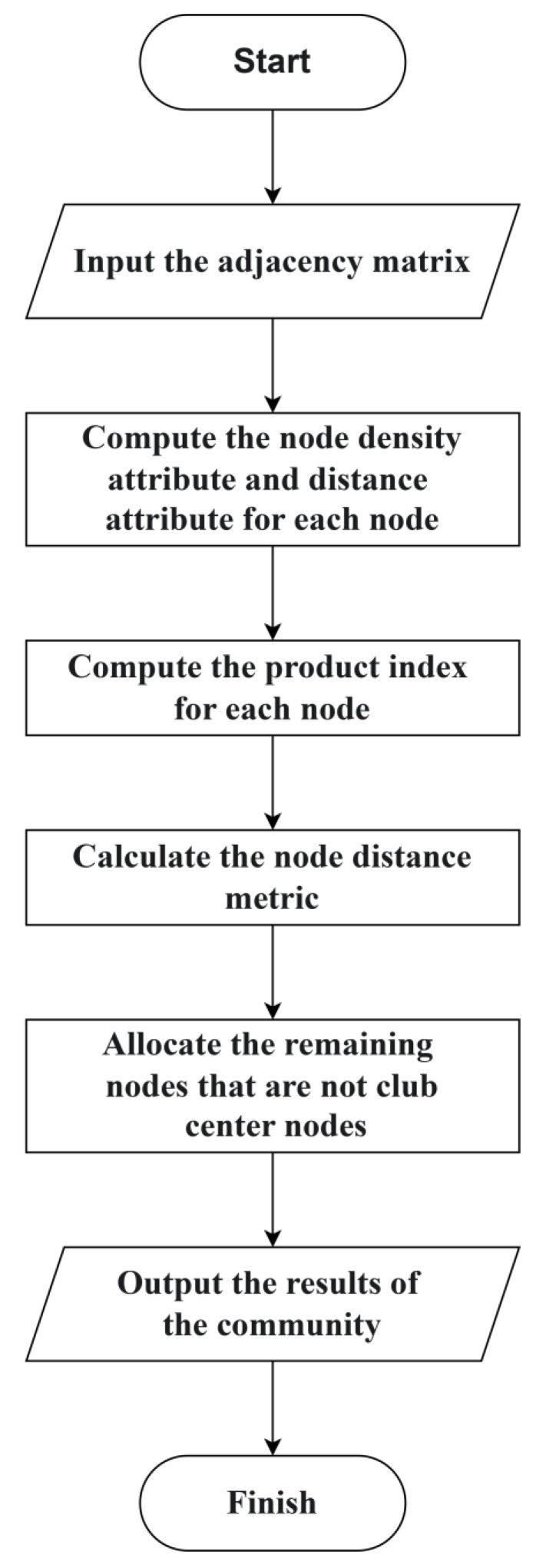
Flowchart of D-means algorithm.

Subsequently, the eigenvectors of the normalized Laplacian matrix are used as the distance metric to compute the similarity between each node and the central nodes. Here, the number of eigenvectors is set equal to the number of community centers, which was automatically determined in the preceding step. Each node is then assigned to the closest community until all nodes have been classified. The overall workflow of the D-means algorithm is illustrated in Figure 3.

The detailed procedure is described as follows:

Input: Complex network G=(V,E).

Output: Results of community division.

Step 1: Calculate the node density $\rho_{i}$ and distance attributes $\delta_{i}$ of each node, and normalize these attributes;

Step 2: Calculate the product index $\gamma_{i}=\rho_{i}^{nor}\times \delta_{i}^{nor}$ of each node and set an upper limit using the Chebyshev inequality to automatically select the center point of the community;

Step 3: Use the eigenvectors of the standardized Laplacian matrix L as the distance metric;

Step 4: Calculate the distance between each node and the center of each community, and assign the node to the center of the community with the closest distance;

Step 5: The algorithm stops immediately after the final assignment of nodes to communities.

### 3.4. Analysis of Algorithm Complexity and Scalability

Assuming a network with N nodes and M edges, the time complexity of computing node density attributes—which incorporate both node degree and clustering coefficient—is O(N+M). The computation of node distance attributes also requires $O(N^{2})$. Normalization involves processing each element, resulting in a complexity of O(N). The identification of community center nodes using Chebyshev inequality has a complexity of O(N). The eigenvalue and eigenvector computation of the Laplacian matrix dominates this process with a time complexity of $O(N^{3})$. Considering all steps, the overall time complexity of the proposed D-means algorithm is $O(N^{3})$. Due to the high complexity arising from the need for precise eigenvector computation, the algorithm’s capability to process ultra-large-scale networks is constrained. Therefore, D-means is currently more suitable for small- to medium-scale network analysis scenarios that place a high demand on automatic center identification.

## 4. Algorithm Validation and Analysis

To evaluate the performance of the proposed algorithm, this paper conducts comparative experiments between the D-means algorithm and six classical community detection methods: GN (Girvan–Newman algorithm), FN (fast community detection algorithms), Louvain (Louvain method for community detection in large networks), Walktrap (Walktrap algorithm for community detection using short random walks), K-means (K-Means-based Community Detection Algorithm), and LPA (Label Propagation Algorithm, LPA). The test datasets include LFR benchmark networks and four widely used real-world networks. Multiple evaluation metrics, such as ACC, NMI, Q, ARI and F1 (F1-Score) are employed to assess the accuracy of the community partitions. Each algorithm was independently executed 10 times on each dataset, and the final results are reported as the average values of each metric to ensure stability and reliability.

### 4.1. Datasets and Parameter Settings

All experiments were conducted in a Python 2021 environment, with results visualized using Origin 2022 software. The evaluation utilized both real-world networks and LFR benchmark datasets. For the LFR networks, algorithm accuracy was assessed by varying key parameters, with initial configurations set as follows: K=10, $max_{K}=60$, mu=0.1, $t_{1}=2$, $t_{2}=1$, $min_{c}=200$, $max_{c}=400$. Table 1 summarizes the specifications of the real and synthetic networks used in this study, where C denotes the number of ground-truth communities, N the number of nodes, M the number of edges, K the average degree, and mu the mixing parameter. In the table, “-” indicates that the corresponding parameter does not need to be set.

### 4.2. Evaluation Metrics

In order to comprehensively describe the reliability and effectiveness of the algorithm, we used five evaluation metrics, including Q, ACC, ARI, NMI, and F1. Their meanings and specific calculation formulas are as follows:

Q denotes the modularity, Assuming the network is partitioned into k communities, the formula for Q is:

(16) $$Q=\sum_{i=1}^{k}\left(\frac{e_{ii}}{m}-{\left(\frac{a_{i}}{2m}\right)}^{2}\right)$$

In the formula, $e_{ii}$ represents the number of internal edges within community i, $a_{i}$ represents the sum of the degrees of all nodes in community i, and m represents the total number of edges in the entire network. The value of modularity ranges between −1 and 1. A value closer to 1 indicates denser connections within communities, implying a more pronounced community structure.

ACC represents the proportion of nodes correctly partitioned by the algorithm. Assume the true community structure of the network is $A=\{A_{1},A_{2},\ldots ,A_{k}\}$, and the community structure computed by the algorithm is $B=\{B_{1},B_{2},\ldots ,B_{k}\}$, where k represent the number of communities. The formula for ACC is:

(17) $$\mathrm{ACC}=\frac{N_{corrent}}{N}$$

In the formula, $N_{corrent}$ refers to the number of nodes for which the community detected by the algorithm exactly matches the true community structure. N is the total number of nodes. The value of ACC ranges from 0 to 1. A larger value indicates a more accurate partition.

ARI denotes the adjusted rand index, The formula for ARI is:

(18) $$\mathrm{ARI}=\frac{\mathrm{RI}-E[\mathrm{RI}]}{\max(\mathrm{RI})-E[\mathrm{RI}]}$$

In the formula, RI is the Rand Index, E[RI] is its expected value, and max(RI) denotes the maximum possible value of RI. The value of ARI ranges from −1 to 1. A value closer to 1 indicates a higher consistency in the community partition, while a value closer to 0 or −1 indicates lower consistency.

NMI denotes Normalized Mutual Information. Assume A and B represent the true community structure and the algorithmically detected community structure, respectively. The formula for calculating NMI is:

(19) $$\mathrm{NMI}(A,B)=\frac{2I(A,B)}{H(A)+H(B)}$$

In the formula, I(A,B) represents the mutual information between A and B, while H(A) and H(B) denote the entropy of A and B, respectively.

The value of NMI ranges from 0 to 1. A larger value indicates that the community structure obtained by the algorithm is closer to the ground-truth structure. When NMI = 1, it signifies that the algorithm’s result is completely consistent with the true community partition. Conversely, when NMI = 0, it indicates that the partition obtained by the algorithm is entirely independent of (or completely different from) the true community partition.

F1 denotes the F1-Score, The F1-Score for community detection is calculated by treating the problem as the classification of node pairs. First, we count the number of node pairs that are correctly grouped together in both the ground-truth and detected partitions (True Positives, TP), as well as the misclassified pairs (False Positives, FP and False Negatives, FN). Then, Precision (P=TP/(TP+FP)) and Recall (R=TP/(TP+FN)) are computed. Finally, the F1-Score is derived as their harmonic mean: F1=2·(P·R)(P+R).

### 4.3. Automatic Selection of Central Nodes

**Figure 4:**
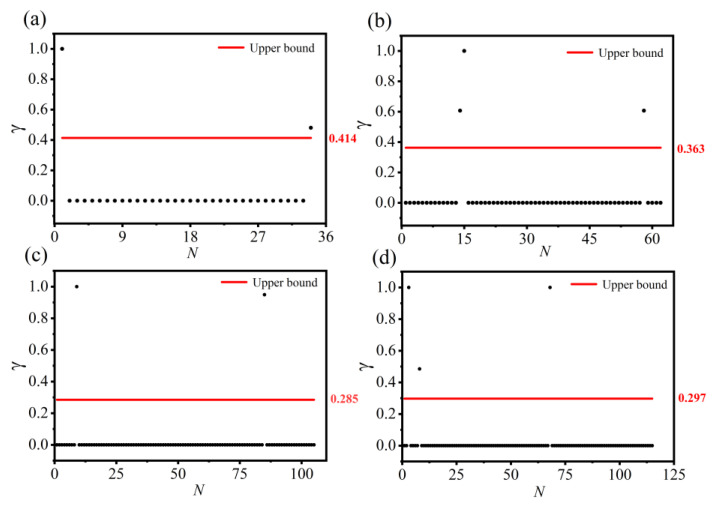
Automated identification of community center nodes on four real-world networks: (**a**) Karate; (**b**) Dolphins; (**c**) Polbooks; (**d**) Football.

Figure 4 illustrates the method for determining community center nodes using Chebyshev’s inequality. Each subplot displays the product index γ of different network nodes, with the horizontal axis representing the node index and the vertical axis representing the value of γ. The red line indicates a preset upper threshold. Any node whose γ value exceeds this threshold is identified as a community center node. As shown in the subplots, the γ values of the identified center nodes are significantly higher than those of ordinary nodes, demonstrating a clear separation between center nodes and others. In the Karate network, for example, the threshold is set to γ=0.414, and Node 1 and Node 34 are automatically selected as center nodes. For comparison, the traditional density peak clustering algorithm—which relies on visual inspection—also identifies the same two center nodes. This indicates that the centers selected by the Chebyshev-based method are highly consistent with those chosen manually. This approach reasonably sets the threshold and effectively identifies center nodes without increasing computational complexity, thereby overcoming the subjectivity and high computational cost of traditional methods.

### 4.4. Comparative Analysis of Algorithms

(1) Experiments on real datasets

This section will use the real network dataset in Table 1 above to test the D-means algorithm. The performance of the algorithm will be evaluated by comparing and analyzing the evaluation indicators of each dataset. Due to the applicability of the algorithm, the symbol “-” in the table indicates that the relevant data values are extremely small (approximately zero), rendering them insignificant for comparison purposes.

Table 2 compares the evaluation metrics of the D-means algorithm against six other methods on real-world datasets. The results demonstrate that D-means achieves better overall performance in terms of ACC, ARI, and NMI. However, it does not excel in modularity, as the algorithm does not rely on modularity optimization for network partitioning.

**Figure 5:**
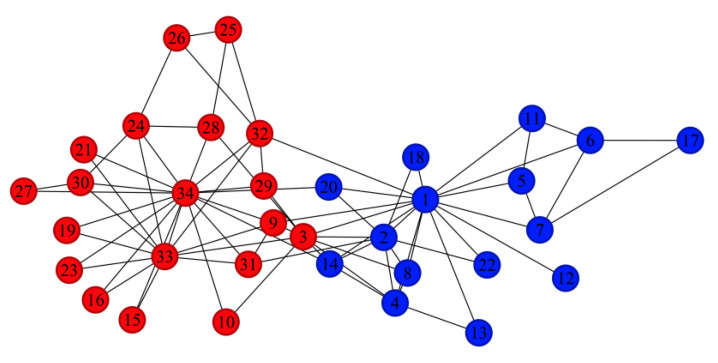
Community structure identified by the D-means algorithm in the Karate network (Numbers represent different nodes in the network, and different colors represent different communities).

On the Karate dataset, the ACC, ARI, and NMI of D-means are significantly higher than those of other algorithms and closely align with the results of K-means, whereas the performance of other methods is considerably lower. Figure 5 visualizes the partitioning result of D-means on the Karate network. Only one node (Node 3) is misclassified, which can be attributed to its position at the community boundary and its strong connections to nodes in both communities, leading to ambiguity in community assignment.

**Figure 6:**
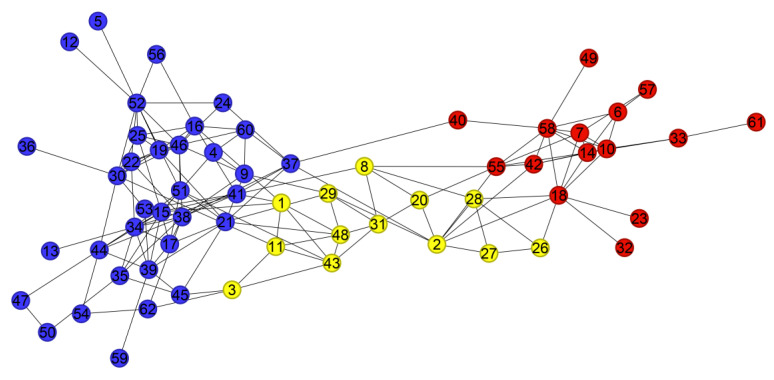
Community structure identified by the D-means algorithm in the Dolphins network (Numbers represent different nodes in the network, and different colors represent different communities).

For the Dolphins dataset, the metrics of D-means are slightly lower than those of K-means but remain superior to other algorithms. The ground-truth network contains two communities, while D-means identifies three, suggesting that the algorithm captures a more fine-grained community structure. The corresponding partition result is shown in Figure 6.

**Figure 7:**
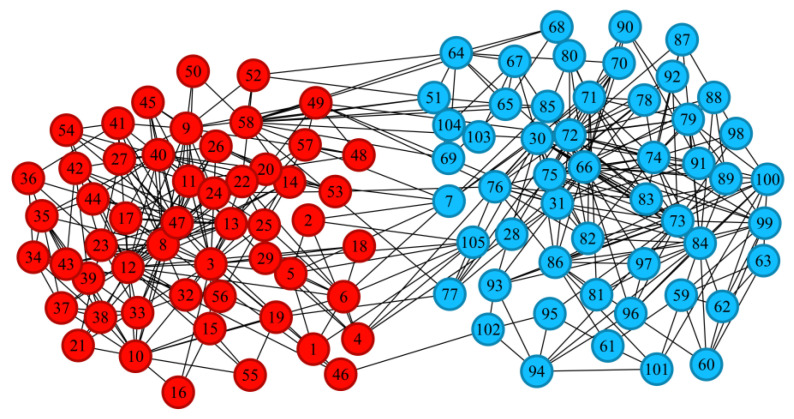
Community structure identified by the D-means algorithm in the Polbooks network (Numbers represent different nodes in the network, and different colors represent different communities).

**Figure 8:**
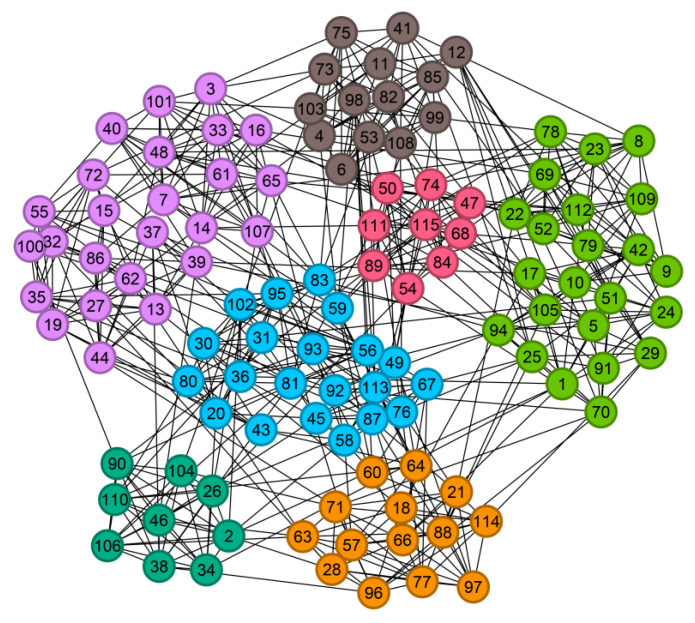
Community structure identified by the D-means algorithm in the Football network (Numbers represent different nodes in the network, and different colors represent different communities).

Figure 7 shows the partitioning results of the D-means algorithm on the Polbooks dataset. The real Polbooks network is divided into three communities, and the D-means algorithm only identifies the center points of two communities. Through further analysis of the internal community structure, it was found that the unrecognized community only contains 13 nodes, and the distance attributes of these nodes are small, which makes it impossible to identify them as an independent community. Although there is some error, the overall performance of this algorithm is still superior to other algorithms. Figure 8 shows the performance of the D-means algorithm on the Football dataset. On this dataset, the K-means algorithm performs the best, while the D-means algorithm’s partitioning results are not ideal. Only 7 communities were identified on this dataset using the D-means algorithm, while the actual number of communities was 12. The reason for this phenomenon is that there are potential community center points in the communities that are directly connected to another community node with a higher density attribute, resulting in the community center points being recognized as ordinary nodes, which affects the division of communities.

(2) Experiments on artificial benchmark network

The GN algorithm suffers from high computational complexity, particularly in large-scale networks where its efficiency decreases exponentially. Consequently, it was excluded from subsequent tests on synthetic benchmark networks. Table 3 compares the performance of the D-means algorithm against five other methods on artificial benchmark networks. Due to the applicability of the algorithm, the symbol “-” in the table indicates that the relevant data values are extremely small (approximately zero), rendering them insignificant for comparison purposes. Experimental results demonstrate that D-means achieves superior community detection performance, confirming its effectiveness.

Among the other algorithms, K-means delivers the strongest overall results but relies on a predefined number of communities, limiting its practical applicability. The D-means algorithm performs closely behind K-means across all metrics, with only marginal differences, yet does not require prior knowledge of the community count—a significant advantage. The Louvain algorithm executes rapidly and scales well to large networks, though it is outperformed by D-means on both the LFR1 and LFR2 datasets. The Walktrap algorithm, based on random walks, is sensitive to the starting node and random seeds, and its performance depends heavily on parameters such as walk length. This necessitates extensive tuning, increasing the practical cost of application. LPA performs poorly on large-scale networks or complex structures, exhibiting strong randomness and poor robustness. In contrast, the FN algorithm performs the weakest, as it incorporates numerous redundant node queries during computation, leading to significantly lower evaluation scores and the poorest partitioning accuracy among all compared methods.

Table 4 compares the execution time of the D-means algorithm against five other methods on synthetic benchmark networks. Experimental results indicate that D-means achieves the highest time efficiency, with its advantage becoming more pronounced as network size increases. The K-means algorithm ranks second in speed, demonstrating high efficiency on smaller networks, though its runtime grows rapidly with increasing node count. While Louvain performs well in detection accuracy, its execution time exceeds that of D-means by more than threefold. LPA exhibits low time complexity with gentle growth scaling. The Walktrap and FN algorithms show the highest computational cost, with runtimes over 200 times greater than that of the D-means method. When the D-means algorithm is executed on a benchmark network with 10,000 nodes, its runtime significantly increases, indicating that the algorithm is currently unsuitable for handling large-scale network computation tasks.

In summary, the D-means algorithm requires no parameter tuning across different networks, demonstrating strong stability and high efficiency in most scenarios, which significantly enhances its flexibility and practical applicability. By integrating the advantages of both DPC and K-means algorithms, it provides an automated and robust community detection solution for small- to medium-scale networks.

## 5. Application of D-Means Based Community Detection Algorithm in Public Transportation

As a core component of urban infrastructure, the public transportation system exhibits complex structural characteristics. Traditional planning methods are often limited to localized and static analyses, making it difficult to reveal the intrinsic patterns of such systems from a mesoscopic perspective. In this study, we employ the D-means algorithm to investigate the community structure of public transportation networks from a complex network perspective, with the aim of uncovering latent community patterns and functional modules. This approach provides a scientific basis for optimizing urban transportation systems and enhancing public mobility.

**Figure 9:**
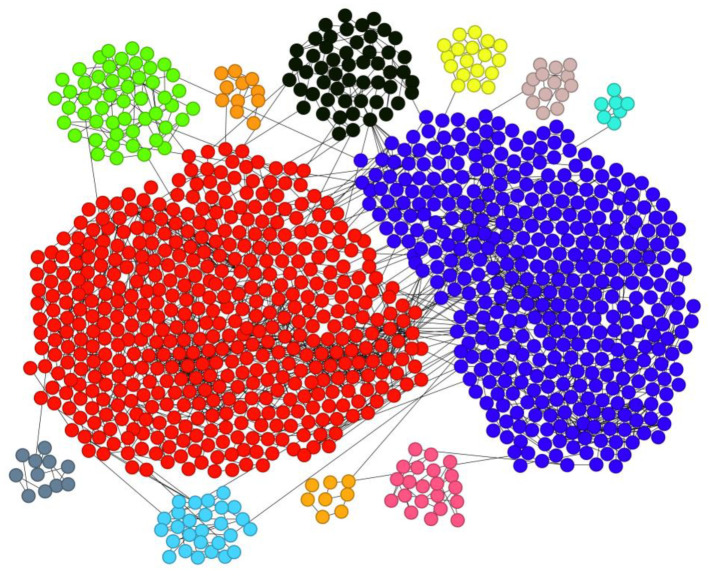
The Community Division Results of MT Network (Different colors represent different communities).

We take the Urumqi Metro-Bus Multimodal Transportation Network (MT Network) as the research object. Applying the D-means algorithm for community detection, we obtain a modularity value of 0.555 and identify 12 communities in the MT network, as visualized in Figure 9. The largest community comprises 434 nodes, while the smallest consists of only 7 nodes.

The city is surrounded by mountains to the east, west, and south, while the northern area consists of a flat alluvial plain formed by the Toutun River. This topography gives the city a loggia-like layout, elongated in the north–south direction and relatively narrow from east to west. Analysis of geographical distribution reveals that the public transportation network exhibits a radial pattern, with density gradually decreasing from the urban center toward the periphery. Tianshan District features the highest concentration of stations, while Urumqi County shows a notably sparser distribution. This spatial disparity reflects significant differences in the functional importance and travel demand across zones within the transportation network. The distribution of stations is influenced by multiple factors, including terrain, economic development, and population density. For instance, economically developed and densely populated areas require a higher density of transit stations to meet residents’ mobility needs, whereas regions with complex terrain or lower population density naturally exhibit a more dispersed station layout.

Larger communities consist of densely connected nodes with frequent interactions, forming tightly knit structural units. The most extensive community, for instance, radiates from key hubs such as Hongqi Road West, Zhongquan Square, Heping North Road, and Qizhong. Centered around commercial districts, it encompasses multiple administrative centers including the Market Supervision Bureau, Tax Bureau, Regional Government, and the Xinjiang Branch of the National Financial Regulatory Administration, collectively forming a closely interconnected functional zone focused on commerce and administration. The strong linkages among these nodes facilitate not only commercial exchanges but also enhance social interactions within the region. Another community, centered on Henan East Road in the new urban area, spans multiple residential complexes such as the 23rd Street Residential Community, Chenxin Residential Community, and Zhongtian Hanlin Finished Homes, while also incorporating major scientific and educational institutions including Xinjiang University of Finance and Economics, Xinjiang Vocational University, Xinjiang Police College, and the Chinese Academy of Sciences (Xinjiang Branch). This integration reflects a distinct atmosphere characterized by academic activity and residential life. The compact spatial structure of these communities is often situated in areas with high transportation density in Urumqi. Due to topographical constraints such as rivers and southern mountains, industrial emissions tend to accumulate in the northern plains, leading to the concentration of heavy industrial zones in the northern part of the city. This is exemplified by the deep orange community, which centers around the Shayibak District Corps Industrial Park. Smaller communities, though comprising fewer nodes, maintain strong internal connections and often represent relatively independent or peripheral units within the network. For instance, the light yellow community is located in the industrial park of the new district, clustering enterprises such as China National Pharmaceutical Group Xinjiang Special Pharmaceutical Co., Ltd., Laiwo Technology, and Xinjiang Zhongtai Special Power Equipment Co., Ltd., indicating a clear correlation between community formation and industrial-economic activities. Similarly, the blue-green community in eastern Shuimogou District contains multiple large recreational venues. Despite scattered transportation routes, internal connectivity remains strong. Community division within the urban public transportation network is shaped by a combination of topographic, economic, and social factors. This is reflected in the spatial distribution of communities such as the pink, black, and brown ones, located, respectively, in residential areas of Urumqi County, Shuimogou District, and Toutunhe District, highlighting the role of community structure in serving daily living and residential needs.

The analysis reveals that stations within the same community are geographically adjacent and strongly interconnected. The urban network exhibits clear functional zoning: Tianshan District is dominated by commercial and administrative areas, the new urban area by research-oriented residential clusters, the northern new district by industrial parks, Shuimogou District by large entertainment venues, and Toutunhe and Midong Districts by industrial development. The public transportation layout is shaped by topography, urban functional zoning, economic development, and population distribution. To enhance regional economic vitality, urban planning should prioritize the placement of major facilities such as shopping malls, hospitals, and schools near central nodes. Bridging nodes, which connect multiple communities, are critical to network resilience; their failure or congestion could disrupt inter-community traffic flow. The D-means algorithm effectively uncovers the community structure of the city’s public transportation network, reflecting its structural and functional characteristics, and provides robust support for urban planning and traffic management.

## 6. Conclusions

In this study, the density peak clustering algorithm is applied to community structure detection, addressing the limitations of the traditional K-means algorithm. A density peak-based community detection algorithm, termed D-means, is proposed by integrating and refining the K-means framework. This algorithm redefines node density and distance attributes to enable the automatic identification of central nodes and determination of the number of communities. To evaluate its performance, longitudinal experiments were conducted on benchmark networks and real-world datasets for comparative analysis. The results demonstrate that the D-means algorithm exhibits stable and satisfactory performance, with significantly reduced running time compared to existing methods. Furthermore, the algorithm was applied to partition the urban transportation network of Urumqi. The analysis revealed that stations within the same community are geographically proximate and highly interconnected, while central nodes exert a considerable influence on both traffic flow and economic dynamics within the community. Influenced by factors such as terrain, economic activity, and industrial layout, the network communities display distinct regional and functional characteristics. These findings indicate that the D-means algorithm can effectively identify community structures and functional zones in urban transportation networks. With technological advancements, real-world networks have grown increasingly large in scale and often exhibit overlapping community structures. However, due to its reliance on eigendecomposition, this algorithm suffers from high computational complexity, making it unsuitable for large-scale network detection and incapable of identifying overlapping communities. To enhance the algorithm’s scalability, we plan to adopt approximation techniques such as the Lanczos algorithm and iterative methods in subsequent work to reduce computational complexity. Furthermore, after spectral embedding, we will replace K-means with C-means clustering for soft assignment, thereby extending the algorithm to handle overlapping communities.

## Figures and Tables

**Table 1:** Real Network Dataset and Synthetic Network Parameters.

Name	C	N	M	K	mu
Karate	2	34	78	4.59	-
Dolphins	2	62	159	5.13	-
Football	12	115	613	10.66	-
Polbooks	3	105	441	8.4	-
LFR1	3	1000	5336	10	0.1
LFR2	6	2000	10,646	10	0.1
LFR3	10	3000	15,822	10	0.1
LFR4	14	4000	21,289	10	0.1

The “-” in the table indicates that this parameter is not applicable to the network.

**Table 2:** Comparison of D-means algorithm with six other algorithms on real datasets.

	Karate					Dolphins				
	Q	ACC	ARI	NMI	F1	Q	ACC	ARI	NMI	F1
GN	0.401	0.441	0.469	0.580	0.588	0.519	0.113	0.395	0.554	0.363
FN	0.381	0.176	0.271	0.243	0.530	0.492	0.355	-	0.004	0.405
Louvain	0.389	0.647	0.645	0.678	0.753	0.510	0.097	0.318	0.507	0.325
Walktrap	0.400	0.765	0.229	0.189	0.506	0.501	0.694	-	0.017	0.258
K-means	0.360	0.971	0.882	0.836	0.883	0.379	0.984	0.935	0.889	0.538
LPA	0.372	0.971	0.882	0.837	0.971	0.384	0.403	−0.012	0.022	0.273
D-means	0.360	0.971	0.882	0.836	0.562	0.444	0.887	0.567	0.522	0.651
	**Polbooks**					**Football**				
	**Q**	**ACC**	**ARI**	**NMI**	**F1**	**Q**	**ACC**	**ARI**	**NMI**	**F1**
GN	0.517	0.810	0.682	0.558	0.659	0.600	0.296	0.778	0.879	0.138
FN	0.502	0.067	0.638	0.531	0.736	0.568	0.078	0.002	0.141	0.918
Louvain	0.518	0.076	0.631	0.555	0.641	0.602	-	0.616	0.813	0.160
Walktrap	0.526	0.848	0.665	0.554	0.653	0.603	0.226	-	0.213	0.567
K-means	0.499	0.838	0.675	0.574	0.716	0.601	0.913	0.897	0.924	0.108
LPA	0.477	0.829	0.613	0.537	0.689	0.579	0.174	0.789	0.889	0.195
D-means	0.512	0.848	0.546	0.500	0.558	0.583	0.635	0.565	0.771	0.475

The “-” in the table indicates that the algorithm is not applicable, leading to poor partitioning results with values close to zero, which are meaningless.

**Table 3:** Comparison of D-means algorithm and four other algorithms on artificial benchmark network.

	LFR1					LFR2				
	Q	ACC	ARI	NMI	F1	Q	ACC	ARI	NMI	F1
FN	0.564	0.328	-	0.002	0.334	0.722	0.167	-	0.003	0.170
Louvain	0.554	0.972	0.916	0.875	0.730	0.730	-	0.998	0.997	0.997
Walktrap	0.565	0.356	-	0.0015	0.730	0.730	0.215	-	0.003	0.170
K-means	0.566	1	1	1	1	0.730	1	1	1	1
LPA	0.556	0.347	1.0	1.0	0.333	0.730	0.686	0.997	0.997	0.666
D-means	0.561	0.999	0.978	0.971	0.985	0.692	0.987	0.914	0.922	0.927
	**LFR3**					**LFR4**				
	**Q**	**ACC**	**ARI**	**NMI**	**F1**	**Q**	**ACC**	**ARI**	**NMI**	**F1**
FN	0.798	0.093	-	0.006	0.101	0.821	0.115	-	0.008	0.075
Louvain	0.799	-	1	1	1	0.825	-	1	1	1
Walktrap	0.799	0.135	-	0.0579	0.101	0.822	0.116	0.001	0.008	0.075
K-means	0.799	1	1	1	1	0.825	1	1	1	1
LPA	0.799	0.223	1.0	1.0	0.2	0.824	0.105	0.998	0.998	0.071
D-means	0.775	0.998	0.970	0.997	0.972	0.778	0.996	0.924	0.958	0.930

The “-” in the table indicates that the algorithm is not applicable, leading to poor partitioning results with values close to zero, which are meaningless.

**Table 4:** Running time results of five algorithms on artificial benchmark networks.

Time/s	LFR1	LFR2	LFR3	LFR4
FN	53.18	402.48	1403.76	3480.95
Louvain	3.445	10.14	11.27	35.38
Walktrap	203.02	2558.94	34,189.70	38,734.87
K-means	0.828	2.836	7.279	49.19
LPA	9.003	9.350	9.519	10.089
D-means	0.984	3.025	7.231	14.12

## Data Availability

The original data used in this study are sourced as follows: The four network datasets (Karate, Dolphins, Football, Polbooks) are publicly available with the kind permission of Professor Mark Newman from the Department of Physics and the Center for the Study of Complex Systems at the University of Michigan, and are stored at https://public.websites.umich.edu/~mejn/netdata (accessed on 5 June 2025); The LFR benchmark dataset is generated based on the literature: Lancichinetti, A., Fortunato, S., & Radicchi, F. (2008). Benchmark graphs for testing community detection algorithms.Physical Review E, 78 (4), 046110 [23]; Urumqi Metro-Bus Multimodal Transportation Network dataset will be provided as Supplementary Materials.

## Supplementary Materials

The following supporting information can be downloaded at: https://www.mdpi.com/article/10.3390/e28020152/s1.
